# Racism, discrimination, and distress: A cross-sectional examination of smoking behaviors among adults who smoke during the COVID-19 pandemic

**DOI:** 10.18332/tpc/215707

**Published:** 2026-03-12

**Authors:** Robert T. Fairman, Dennis E. Reidy, Lucy Popova, Claire A. Spears

**Affiliations:** 1 Department of Health Promotion and Physical Education, Wellstar College of Health and Human Services, Kennesaw State University, Kennesaw, United States; 2 School of Public Health, Georgia State University, Atlanta, United States

**Keywords:** health disparities, tobacco use, COVID-19, mental health

## Abstract

**INTRODUCTION:**

Racial and ethnic disparities in tobacco use and related health consequences persist. Experiences with racial discrimination have been associated with higher likelihood of smoking and worse cessation outcomes. The purpose of this study is to examine associations between perceived discrimination with serious quit attempts, smoking relapse, and changes in motivation to quit smoking, during the COVID-19 pandemic.

**METHODS:**

Data came from an October–November 2020 US nationally representative cross-sectional survey of adults who currently smoked cigarettes (n=1223). Participants reported changes in smoking frequency, motivation to quit, quit attempts, and relapse since February 2020. Perceived discrimination was assessed using a modified Everyday Discrimination Scale, the Complementary and Integrative Research Lab Pandemic Impact Questionnaire, and Domain-Specific Stress Scales. In addition to the associations between discrimination and smoking behaviors, we explored differences in smoking behaviors by race.

**RESULTS:**

Approximately 13–25% of participants reported having relapsed to smoking since the beginning of the COVID-19 pandemic. Black participants perceived higher levels of everyday discrimination and were more likely to report having been avoided or insulted because of their race or ethnicity. Participants who experienced racism and discrimination were more likely to have made a serious attempt to quit smoking since the beginning of COVID-19 (AOR=1.80; 95% CI: 1.08–3.03). Experiences with police brutality (AOR=1.61; 95% CI: 1.31–1.97), as well as being avoided or insulted because of their race or ethnicity (AOR=4.70; 95% CI: 2.75–8.04) was associated with higher likelihood of relapse.

**CONCLUSIONS:**

Perceived experiences with racism and discrimination among people who smoke were associated with making a serious smoking quit attempt but also predicted relapse. Further research can develop smoking cessation interventions that consider various social stressors, such as experiences with racism and discrimination.

## INTRODUCTION

Experiences with racism and discrimination are associated with deleterious mental and physical health outcomes across various populations including racial and ethnic minoritized groups, as well as those identifying as non-Hispanic White^1-4^. Discrimination can occur on a day-to-day basis, such as perceiving that one has been treated with less courtesy or respect than other people, as well as related to major life events such as being unfairly fired from a job or unfairly stopped, questioned or threatened by the police. Experiences with discrimination may be attributed to various factors such as one’s race, ancestry, gender, age, religion, weight, sexual orientation, and others^5,6^. Perceived discrimination has been linked to various types of substance use and abuse, including higher cigarette smoking and lower smoking cessation rates across various racial and ethnic groups including minoritized populations and non-Hispanic Whites^4,7-9^.

Smoking and other substance use can be an avoidant coping strategy in the context of stressful experiences of discrimination^10^. Stress and difficulty regulating negative emotions have been noted as some of the key drivers of tobacco use. People with multiple minoritized statuses, such as race, ethnicity, gender, or sexual orientation, may experience greater exposure to minority stressors, exacerbating their tobacco use severity^10,11^. Additionally, those who experience greater stressful life events are more likely to have moderate to severe tobacco use disorder, compared to those who experience lower levels of stressful life events^10^. Several studies suggest that psychological distress mediates the association between perceived discrimination and smoking, such that distress associated with experiences of discrimination triggers smoking behavior^12-14^.

In the US and across the world, the COVID-19 pandemic has been a period of heighted stress and psychological distress, and the health effects of the pandemic have been exacerbated by poverty and racial discrimination^15^. This has also been a time of heightened awareness of racism and hate crimes against minoritized communities^16,17^. During the COVID-19 pandemic, experiences with racial discrimination have been associated with higher psychological distress and cigarette smoking^16^. Moreover, greater concern about COVID-19 discrimination has been linked to higher cigarette consumption and greater odds of smoking relapse among adults who smoke cigarettes^18^.

There has been much variability in smoking patterns in the context of the pandemic, with some people smoking more, others smoking less, and others reporting no change^19^. For those who smoked more, this seemed to be fueled, at least in part, by attempts to manage stress, anxiety, and other negative emotions^20^. Some studies have shown that compared to Whites, Black adults had higher intentions to quit smoking and were more likely to have attempted to quit smoking during COVID-19, which may be due to being at higher risk of complications and death due to COVID-19^21^. Since those who smoke are at heightened risk for COVID-19 progression, as are minoritized populations such as those who identify as Black or Hispanic/Latinx and those dealing with negative emotions, which impedes their ability to quit smoking, research is needed to further examine changes in smoking behaviors among racial and ethnic minoritized populations^22-24^. This study aimed to examine whether discrimination, racism, and/or experiences with social injustice were associated with smoking behaviors among those who smoked cigarettes during the COVID-19 pandemic. We hypothesized that those who reported experiences with racism, discrimination, and injustices would be more likely to relapse, and also less likely to make serious tobacco use quit attempts.

## METHODS

### Study sample and procedures

Data were drawn from a 2020 (October–November) cross-sectional survey of a US national probability sample drawn from Ipsos Public Affairs’ KnowledgePanel, a probability-based web panel designed to be representative of non-institutionalized US adults. Panel members were provided incentives for participation in the panel and could enter raffles for prizes^25^. Adult panelists (18 years and older) who reported current cigarettes smoking or current electronic nicotine delivery system (ENDS) use on their recent Ipsos profile, were sampled randomly and invited to participate once they confirmed currently smoking cigarettes (defined as having smoked at least 100 cigarettes in their life, and now smoking either ‘every day’ or ‘some says”) or used ENDS (defined as using “some days’ or ‘every day’), or had recently quit using cigarettes or ENDS (since February 2020). Among the 2752 KnowledgePanel participants that were invited, 55% (n=1526) completed the survey. Those who did not currently use cigarettes (including not having used in the past 30 days) were excluded from the present analysis, resulting in a final analytic sample of 1223 people who currently smoked. While the overall study included people who smoked and/or used ENDS, this report focused on smoking behaviors. A study-specific post-stratification weight was computed using an iterative proportional fitting (raking) procedure using benchmarks from 2019 National Health Interview Survey (gender, race/ethnicity, census region, metropolitan status, education)^26^ and KnowledgePanel profile information (household income) to account for sources of sampling and non-sampling errors. These errors included attrition, panel recruitment non-response, and nonresponse to study invitation. The Georgia State University Institutional Review Board determined that this study was exempt from federal human subjects research regulations.

### Measures

*Independent variables*

Sociodemographic variables included age (18–29, 30–44, 45–59, ≥60 years), race/ethnicity (White, non-Hispanic; Black, non-Hispanic; Other, non-Hispanic; Hispanic), education (lower than high school, high school, some college, Bachelor’s degree or higher), annual household income ($) (<30000, 30000–99999, and >100000), employment status (working; not working- laid off/looking for work, not working- retired, not working- disabled, not working- other), health insurance status (private or insured, Medicare, Medicaid, VA, Dept of Defense or Military Program, uninsured), gender (male, female), gender identity (cisgender, transgender, other), and sexual orientation (straight, other). A modified Everyday Discrimination Scale (EDS short version) was used to assess perceived racial/ethnic discrimination, utilizing a five-point Likert-scale, ranging from ‘never’ to ‘almost every day’^5^. The opening language of the Everyday Discrimination Scale was modified to specifically assess discrimination attributed to race or ethnicity in the context of the pandemic: ‘In your day-to-day life, how often have any of the following happened to you because of your race or ethnicity since February 2020 (the start of the COVID-19 pandemic)?’. An example item is ‘You are treated with less courtesy or respect for other people’. Responses on the five items were summed to create a total score. Experiences with unfair treatment by police (attributed to race or ethnicity) were assessed separately using a modified item from the Major Experiences of Discrimination Scale: ‘In your day-to-day life, how often have any of the following happened to you because of your race or ethnicity since February 2020 (the start of the COVID-19 pandemic)? You are unfairly stopped, searched, questioned, physically threatened or abused by the police’^5^. Next, a modified Complementary and Integrative Research Lab Pandemic Impact Questionnaire (C-PIQ, 8 items) was utilized to access events that happened in the past year, as it relates to COVID-19^27^. An example measure includes: ‘People have avoided me or insulted me more often because of my race/ethnicity’ with responses being ‘yes’ or ‘no’. An adapted Domain-Specific Stress Scale (8 items) measured COVID-19-related worries, for example: ‘I am worried about protests or experiences with racism, discrimination, or police injustice’^28^. Responses were dichotomized to indicate agreement with each worry (‘agree’ and ‘strongly agree’ vs all other answers).

*Outcomes*

Relapse to smoking in the context of the early COVID-19 pandemic was measured by asking: ‘Did you quit smoking sometime before February 2020 but then start smoking again after February 2020?’ with response options of yes or no. Frequency of smoking in the past 30 days was assessed using: ‘During the past 30 days, on how many days did you smoke cigarettes?’. The number of cigarettes per day was measured using: ‘On average, on the days that you smoke, how many cigarettes a day do you smoke? A pack usually has 20 cigarettes in it’. Motivation to quit smoking was measured by asking: ‘How has your motivation to quit smoking changed since February 2020?’ with responses ranging from increased motivation, motivation has stayed the same, decreased, or other. Having a serious quit attempt was measured by using: ‘Since February 2020 (the start of the COVID-19 pandemic), have you ever made a serious attempt to quit smoking? That is, have you stopped smoking for at least one day or longer because you were trying to quit?’, with responses being yes or no.

### Data analysis

Unadjusted associations between race/ethnicity and experiences with racism, discrimination, police injustice, and smoking behaviors were conducted using Rao-Scott chi-squared analyses for weighted categorical outcomes and ANOVAs for continuous outcomes. Adjusted associations between experiences of discrimination and smoking behaviors were performed using weighted logistic regression and proportional odds models, controlling only for race and ethnicity. All analyses were conducted using SAS 9.4, with p<0.05 indicating statistical significance.

## RESULTS

This sample included 1223 persons who currently smoked cigarettes (47% female, mean age 52.64 years, SD=14.52). Nearly 70% identified as non-Hispanic White, 13% non-Hispanic Black, 11% Hispanic, and 7% ‘Other, non-Hispanic’. Fifty-five percent had a high school education or less, 47% reported a household income between $30000–99999, 39% were currently not working, 36% were on Medicare or Medicaid, and 14% were uninsured (Table 1).

**Table 1:** Participant characteristics of a nationally representative sample of current smokers in the US in 2020 (N=1223)

	*Unweighted n*	*Weighted % (95%* *CI)*
**Age** (years)		
18–29	73	16.61 (13–20.21)
30–44	293	30.49 (27.2–33.78)
45–59	390	29.66 (26.72–32.6)
≥60	467	23.25 (20.82–25.67)
**Race/ethnicity**		
White, non-Hispanic	920	69.42 (66–72.83)
Black, non-Hispanic	138	13.3 (10.82–15.79)
Other, non-Hispanic	65	6.62 (4.57–8.67)
Hispanic	100	10.66 (8.32–13)
**Education level**		
Lower than high school	111	18.09 (14.87–21.31)
High school	424	37.37 (33.94–40.8)
Some college	476	33.18 (30.05–36.32)
Bachelor’s degree or higher	212	11.35 (9.39–13.31)
**Household income ($)**		
<30000	374	29.69 (26.46–32.93)
30000–99999	619	47.14 (43.64–50.64)
>100000	230	23.16 (19.96–26.37)
**Employment**		
Working	702	60.8 (57.38–64.22)
Not working – laid off/ looking for work	73	9.23 (6.7–11.75)
Not working – retired	298	15.7 (13.61–17.79)
Not working – disabled	110	9.78 (7.8–11.76)
Not working – other	40	4.5 (2.91–6.08)
**Health insurance status**		
Private or insured	527	46.11 (42.55–49.68)
Medicare	302	17.35 (15.03–19.67)
Medicaid	186	18.99 (16.12–21.86)
VA, Dept of Defense or Military Program	57	3.62 (2.38–4.85)
Uninsured	126	13.94 (11.22–16.65)
**Sexual orientation**		
Straight	1169	95.78 (94.18–97.37)
Other	47	4.22 (2.63–5.82)
**Gender**		
Male	678	53.37 (49.86–56.89)
Female	545	46.63 (43.11–50.14)
**Gender identity**		
Cisgender	1201	98.69 (97.9–99.48)
Transgender	8	0.76 (0.14–1.38)
Other	5	0.55 (0.05–1.05)

Based on unadjusted associations between race/ethnicity and smoking behaviors, members of racial/ethnic minoritized groups were more likely to report smoking relapse compared to non-Hispanic Whites (25% among Hispanics, 17% of those identifying as Black, 13% of non-Hispanic Whites; χ^2^=9.1230, p=0.0277) (Table 2). Nearly one-fourth of those who identified as Black reported increased motivation to quit since the beginning of the pandemic. Those who identified as Black also reported a higher proportion of serious quit attempts, compared to non-Hispanic Whites (χ^2^=14.2317, p=0.0026) (Table 2). Non-Hispanic Whites smoked more cigarettes per day (mean=14.00; 95% CI: 13.42–14.58), compared to those who identify as Black (mean=8.86; 95% CI: 7.17–10.55), Hispanics (mean=6.71; 95% CI: 3.27–10.16), and those of other races (mean=12.35; 95% CI: 9.68–15.03, p<0.0001) (Table 3).

**Table 2:** Descriptive statistics of smoking behaviors by race/ethnicity among current smokers in the US in 2020 (N=1223)

*Race/ethnicity*	*Relapse* *Weighted %* *(95% CI)*		*Motivation to quit* *Weighted %* *(95% CI)*			*Serious quit attempt* *Weighted %* *(95 % CI)*	
	*Yes*	*No*	*Increased*	*Stayed the* *same*	*Decreased*	*Yes*	*No*
p	0.02777		0.2991			<0.0001	
White, non-Hispanic	12.56 (9.75–15.37)	87.44 (84.63–90.25)	15.85 (13.11–18.59)	70.2 (66.52–73.88)	13.95 (11.02–16.88)	17.20 (14.20–20.20)	82.80 (79.80–85.80)
Black, non-Hispanic	17.33 (10.45–24.2)	82.67 (75.8–89.55)	24.84 (15.97–33.71)	61.03 (51.1–70.97)	14.13 (7.27–20.98)	32.03 (22.58–41.48)	67.97 (58.52–77.42)
Other, non-Hispanic	12.71 (4.28–21.15)	87.29 (78.85–95.72)	17.22 (6.26–28.17)	64.97 (49.37–80.56)	17.82 (3.94–31.7)	18.17 (6.58–29.76)	81.83 (70.25–93.42)
Hispanic	24.58 (14.28–34.88)	75.42 (65.12–85.72)	17.82 (3.94–31.7)	22.63 (11.57–33.7)	58.24 (46.06–70.43)	29.64 (18.55–40.73)	70.36 (59.27–81.49)

**Table 3:** Mean scores of smoking behaviors by race/ethnicity among current smokers in the US in 2020 (N=1223)

*Race/ethnicity*	*Days smoked cigarettes*	*95% CI*	*Cigarettes per day*	*95 % CI*
p	0.1090		<0.0001	
White, non-Hispanic	14.41	12.99–15.83	14.00	13.42–14.58
Black, non-Hispanic	15.71	12.90–18.53	8.86	7.17–10.55
Other, non-Hispanic	12.46	7.24–17.69	12.35	9.68–15.03
Hispanic	17.56	14.19–20.93	6.71	3.27–10.16

Black participants scored higher on the Everyday Discrimination Scale compared to those of other races and ethnicities. Those who identified as Black averaged a score of 4.54 (SD=5.84) on the Everyday Discrimination Scale, compared to the mean of 1.39 (SD=2.99) among non-Hispanic Whites, 4.27 (SD=6.85) among Hispanics, and 3.91 (SD=6.40) among those of other races (p<0.0001). Those who were Black, Hispanic, or other races reported higher rates of being avoided or insulted because of their race or ethnicity, compared to non-Hispanic Whites. Approximately 35% of those who identified as Black reported having ever been unfairly stopped, searched questioned, physically threatened, or abused by the police because of their race or ethnicity since the start of the COVID-19 pandemic, compared to 25% of Hispanics, 28% of those of other races or ethnicities, and less than 7% of non-Hispanic Whites (p<0.0001) (Table 4).

**Table 4:** Descriptive statistics of discrimination variables among current smokers in a US sample in 2020 (N=1223)

*Race/* *ethnicity*	*Discrimination* *scale* *Items 1–5* *Mean* *(95% CI)*	*Discrimination* *scale (total) ^a^* *Mean* *(95% CI)*	*People have avoided me or* *insulted me more often* *because of my race/* *ethnicity* *Weighted %* *(95% CI)*		*I am worried about protests or* *experiences with racism, discrimination,* *or police injustice* *Weighted %* *(95% CI)*			*In your day-to-day life, how often have any of the following things* *happened to you because of your race or ethnicity since February 2020* *(the start of the COVID-19 pandemic)? You are unfairly stopped,* *searched, questioned, physically threatened or abused by the police* *Weighted %* *(95% CI)*				
			*No*	*Yes*	*Disagree*	*Neutral*	*Agree*	*Never*	*Less than* *once a* *month*	*A few times* *a month*	*At least* *once a week*	*Almost* *everyday*
p	**<0.0001**	**<0.0001**	**<0.0001**			0.7176				0.7176		
White, non-Hispanic	1.29 (1.11–1.47)	1.39 (1.19–1.59)	97.05 (95.54–98.57)	2.95 (1.43–4.46)	29.42 (25.84–33)	30.34 (26.45–4.22)	40.24 (36.35–44.13)	93.28 (91.02–95.54)	4.41 (2.62–6.2)	1.01 (0.23–1.8)	0.91 (0–2.06)	0.38 (0–0.83)
Black, non-Hispanic	4.05 (3.24–4.87)	4.54 (3.63–5.45)	86.97 (79.75–94.2)	13.03 (5.8–20.25)	30.34 (20.67–40.01)	25.24 (17.11–33.36)	44.42 (34.7–54.15)	64.58 (54.47–74.68)	20.69 (11.36–30.03)	12.07 (5.21–18.93)	1.4 (0–3.11)	1.26 (0–3.02)
Other, non-Hispanic	3.37 (2.22–4.52)	3.91\(2.53–5.28)	81.81 (68.89–94.74)	18.19 (5.26–31.11)	29.3 (15.27–43.34)	21.63 (8.46–34.8)	49.06 (33.18–64.95)	76.98 (61.93–92.02)	5 (0–10.04)	8.5 (0–20.14)	6.88 (0–17.47)	2.64 (0–6.64)
Hispanic	3.77 (2.78–4.76)	4.27 (3.1–5.45)	86.66 (77.72–95.6)	13.34 (4.4–22.28)	23.59 (14.18–33)	31.2 (20.52–41.88)	45.21 (33.65–56.78)	71.58 (60.22–82.93)	16.13 (6.88–25.38)	4.05 (0–8.47)	4.73 (0–9.53)	3.52 (0–10.22)

a Discrimination scale total refers to the sum of the modified 6-item Everyday Discrimination Scale, with scores ranging 0–24.

In predicting whether participants had made a serious quit attempt since the beginning of the COVID-19 pandemic, those who experienced discrimination through people avoiding them or insulting them because of their race/ethnicity were more likely to have made a serious quit attempt, compared to those who did not experience that discrimination, while controlling for race and ethnicity (adjusted odds ratio, AOR=1.810; 95% CI: 1.078–3.039, p=0.0249). Those who reported worrying about protests or experiences with racism, discrimination, and police injustice were more likely to have made a serious quit attempt (AOR=1.519; 95% CI: 1.077–2.143, p=0.0172), as well as those who have been unfairly stopped, searched, questioned, physically threatened or abused by the police (AOR=1.289; 95% CI: 1.072–1.549, p=0.0069) compared to those who had not, while controlling for race/ethnicity. For a one unit increase in the Everyday Discrimination Scale, the odds of a serious quit attempt increases by 1.035 times (AOR=1.035; 95% CI: 1.004–1.068, p=0.0280).

Those who had been avoided because of their race/ethnicity were nearly five times more likely to have relapsed to smoking since the beginning of the COVID-19 pandemic (AOR=4.704; 95% CI: 2.753–8.039, p<0.0001), and those who were unfairly stopped, searched, questioned, physically threatened, or abused by the police were almost two times more likely to relapse since the beginning of the COVID-19 pandemic (AOR=1.606; 95% CI: 1.307–1.972, p<0.0001). For a one unit increase in the Everyday Discrimination Scale, the odds of having a relapse increases by 1.074 (AOR=1.074; 95% CI: 1.037–1.113, p<0.0001).

For those who were worried about protests or experiences with racism, discrimination, or police injustice, the odds of quit motivation (increased vs others, or the combined increased and no change vs decreased), was about 0.740 the odds of those who were not worried about protests, racism, discrimination, or police injustice (AOR=0.740; 95% CI: 0.552–0.990, p=0.0428) (Table 5).

**Table 5:** Logistic regression of experiences of racism, discrimination, and injustice predicting smoking behaviors among current smokers in a US sample in 2020 (N=1223)

*Item*	*Since February 2020 (the start of* *the COVID-19 pandemic), have you* *ever made a serious attempt to quit* *smoking? That is, have you stopped* *smoking for at least one day or* *longer because you were trying to* *quit? (Ref. no)*				*Did you quit smoking sometime* *before February 2020 but then* *start smoking again after February* *2020?* *(Ref. no)*				*How has your motivation to quit* *smoking changed since February* *2020? ^a^* *(Ref. decreased)*			
	*AOR*	*95% CI*		*p*	*AOR*	*95% CI*		*p*	*AOR*	*95% CI*		*p*
		*L*	*U*			*L*	*U*			*L*	*U*	
People have avoided me or insulted me more often because of my race/ethnicity (Ref. no)	1.810	1.078	3.039	**0.0249**	4.704	2.753	8.039	**<0.0001**	0.862	0.517	1.438	0.5694
I am worried about protests or experiences with racism, discrimination, or police injustice. (Ref. disagree)	1.519	1.077	2.143	**0.0172**	1.135	0.766	1.681	0.5279	0.740	0.552	0.990	**0.0428**
You are unfairly stopped, searched, questioned, physically threatened or abused by the police (Ref. never)	1.289	1.072	1.549	**0.0069**	1.606	1.307	1.972	**<0.0001**	0.973	0.813	1.164	0.7611
Discrimination sum (one unit increase)	1.035	1.004	1.068	**0.0280**	1.074	1.037	1.113	**<0.0001**	1.023	0.993	1.054	0.1291

Model was adjusted for race and ethnicity.

a Utilized a cumulative ordinal model, presenting results for increased and no change versus decreased.

## DISCUSSION

In this US nationally representative sample of adults who smoke, experiences of racism and discrimination in the context of the COVID-19 pandemic were associated with higher likelihood of having made a serious attempt to quit, but also higher likelihood of relapse to smoking. There is abundant literature supporting the links among discrimination, stress, and tobacco use behaviors^4,8^. This study adds to prior literature by assessing multiple types of perceived discrimination, including police injustice, in the context of the COVID-19 pandemic. Those who reported having been avoided or insulted because of their race/ethnicity, and those who indicated being unfairly stopped, searched, questioned, physically threatened, or abused by the police, were more likely to have relapsed to cigarette smoking.

Negative experiences with the police cause harm, stress, and worsen mental health outcomes^29,30^. Experiences with police discrimination and mistrust have been associated with higher likelihood of heavy episodic drinking and marijuana use^31^. The effects of police brutality on those who are of minoritized status has been named a social determinant of health, because of the direct effect it has on health outcomes^32^. An experience of discrimination can lead to an increased amount of psychosocial stress, which could increase risk for smoking in attempt to cope with the increased stress, which occurred from experiences of discrimination^33^. Those who experience discrimination on a daily basis are more likely to smoke cigarettes compared to those who do not^33^. Further, experiences of everyday discrimination as well as major discrimination were shown to be predictive of regular smoking^34^. Since the COVID-19 pandemic was a time in which experiences of discrimination were highlighted, it is possible that experiences of discrimination increased stress among communities of color, which then increased the likelihood of smoking relapse^35^.

In general, experiences with perceived discrimination have been shown to be associated with lower self-efficacy for smoking cessation^14^. Though various experiences of perceived discrimination were associated with serious quit attempts, previous studies have shown that self-efficacy is a consistent predictor of cessation outcomes^36^. It has also been noted that discrimination lowers self-efficacy, and lower self-efficacy increases the probability of smoking after a quit attempt^36^. This may support the theory that experiencing discrimination may lower one’s confidence to resist smoking during high-risk situations, such as a pandemic, economic uncertainty, feelings of helplessness, or sustained experiences with discrimination.

It is worth noting that in our sample, experiences of perceived discrimination predicted higher likelihood of making a serious quit attempt. It is possible that those who were experiencing perceived discrimination in the face of a global pandemic turned to what they thought they could control – their smoking behaviors.

### Limitations

This study is limited by its cross-sectional design and reliance on self-report, which prevents conclusions related to temporality or causality and raises potential concerns about information bias and misclassification. There is also potential residual confounding (e.g. not all potential confounders were included in the regression models). However, it is strengthened by a large US nationally representative survey of adults who smoke, who are at particular risk for COVID-19-related complications. It is worth noting that approximately 69% of the sample identified as non-Hispanic White, which may not accurately gauge perceptions around experiences of racism, discrimination, or police injustice, and may limit generalizability. Our discrimination measures did not include a healthcare-specific domain (e.g. clinical management or treatment quality). Thus, results pertain to non-clinical contexts (e.g. interpersonal racism, police brutality) and should not be generalized to hospital care. Future studies should incorporate healthcare discrimination measures to assess links with cessation outcomes.

## CONCLUSIONS

Experiences with discrimination in American adults who smoke were associated with higher likelihood of making a serious smoking quit attempt in the context of the COVID-19 pandemic but also predicted higher likelihood of relapse to smoking during this time. Although longitudinal research is needed to clarify the temporality of these associations, it is possible that stressful experiences of racism, discrimination, and injustice can hinder successful smoking cessation. Future qualitative studies could also examine the lived experiences of discrimination to gain a richer understanding of how such experiences might impact individuals’ smoking behaviors. Tailored smoking cessation interventions that consider various social stressors, such as racism and discrimination, among minoritized populations could have value. Ultimately, interventions at the interpersonal, community, and societal levels are needed to combat the health consequences of discrimination by addressing structural and social determinants of health.

## Data Availability

The data supporting this research are available from the authors on reasonable request.
